# Increased risk of varicella-zoster virus infection in patients with breast cancer after adjuvant radiotherapy: A population-based cohort study

**DOI:** 10.1371/journal.pone.0209365

**Published:** 2019-01-09

**Authors:** Yo-Liang Lai, Yuan-Chih Su, Chia-Hung Kao, Ji-An Liang

**Affiliations:** 1 Department of Radiation Oncology, China Medical University Hospital, Taichung, Taiwan; 2 Graduate Institute of Biomedical Sciences, China Medical University, Taichung, Taiwan; 3 Management Office for Health Data, China Medical University Hospital, Taichung, Taiwan; 4 Department of Nuclear Medicine and PET Center, China Medical University Hospital, Taichung, Taiwan; 5 Graduate Institute of Clinical Medical Science, College of Medicine, China Medical University, Taichung, Taiwan; 6 Department of Bioinformatics and Medical Engineering, Asia University, Taichung, Taiwan; Katholieke Universiteit Leuven Rega Institute for Medical Research, BELGIUM

## Abstract

**Background/Purpose:**

Limited evidence has been obtained on varicella-zoster virus (VZV) infection in patients with breast cancer as a complication related to adjuvant radiotherapy. We conducted a cohort study aimed to assess the risk of VZV infection in this patient setting.

**Materials and methods:**

We used the National Health Insurance Research Database to identify 65,981 patients with breast cancer in Taiwan who underwent breast surgery between 2000 and 2011. After a 1:1 propensity score match was obtained between patients with and without radiotherapy, a competing risk regression model was constructed to estimate the hazard ratios and the incidence rate difference (IRD) of VZV infection in the patients with breast cancer receiving radiotherapy and those not receiving radiotherapy.

**Results:**

After adjusting for covariates, the radiotherapy cohort showed a 1.51-fold higher risk (95% confidence interval = 1.06–5.16, p = 0.02, IRD = 4.98/10000 person-years) of subsequent VZV infection than the nonradiotherapy cohort. Furthermore, VZV infection risk was 3.85-fold higher among patients aged >65 years who received radiotherapy than among those of the same age who did not receive radiotherapy (95% confidence interval = 1.1–13.4, p < 0.05, IRD = 11.09/10000 person-years). The risk increased with adjusted hazard ratio of 6.6 (95% confidence interval I = 1.51–28.8, p < 0.05, IRD = 32.01/10,000 person-years) and 7.08 (95% confidence interval = 1.64–30.5, p < 0.01, IRD = 35.72/10,000 person-years) in follow-up period less than 3 months and 3–5 months respectively.

**Conclusion:**

Radiotherapy was associated with an increased risk of VZV infection among patients with breast cancer. The risk was significantly higher in older patient (>65 years old) and/or those who received chemotherapy. Regular clinical follow-up and additional serological testing in the first 5 months after radiotherapy are recommended.

## Introduction

Varicella-zoster virus (VZV) infection, which can cause chicken pox and herpes zoster, usually presents as localized skin disease that shows little preponderance to serious symptoms or sequelae such as neuropathy, vasculopathy, or retinal necrosis [1]. VZV infection risk is associated with compromised immunity due to conditions such as old age, human immunodeficiency virus (HIV) infection, organ transplants, or malignancy-related therapy [2].

Breast cancer is the most common cancer among women worldwide, with more than one million new diagnoses each year [3]. Many patients in this group receive adjuvant radiotherapy (RT), which has been proven to attenuate local recurrence and mortality [4–6]. However, certain side effects can occur during adjuvant RT, such as dermatitis, skin fibrosis, lymphedema, lung toxicity, and heart toxicity [7, 8]. Little information is available on VZV infection in this setting. Only one study evaluated the increased frequency of zoster infection after RT in patients with breast cancer but could not confirm the contribution of RT [9]. In this study, we conducted a nationwide population-based study to elucidate the risk factors for VZV infection in patients with breast cancer receiving adjuvant RT.

## Materials and methods

Patients with breast cancer who underwent breast surgery were identified from the Registry for Catastrophic Illness Patient Database (RCIPD). The RCIPD is a subdatabase of the National Health Insurance Research Database (NHIRD) and contains records of patients with catastrophic illness from 1997 to 2011. The NHIRD contains data from the National Health Insurance program, a single-payer health insurance program mandatory for all citizens (23.74 million residents) with a coverage rate of approximately 99% [10]. The RCIPD contains information about sex, date of birth, clinical visit date, International Classification of Diseases, Ninth Revision, Clinical Modification (ICD-9-CM) codes, and treatment.

The NHIRD encrypts patient personal information to protect privacy and provides researchers with anonymous identification numbers associated with relevant claims information, including sex, date of birth, medical services received, and prescriptions. Therefore, patient consent is not required to access the NHIRD. This study was approved to fulfill the condition for exemption by the Institutional Review Board (IRB) of China Medical University (CMUH104-REC2-115-CR3). The IRB also specifically waived the consent requirement.

We selected patients with breast cancer (ICD-9-CM: 174) who received and did not receive RT during 2000–2010. The RT date was considered the index date of this study. Demographic data only included patients’ age. Chemotherapy and hormone therapy (tamoxifen and letrozole) records after the index date were the treatment variables. History of hypertension (ICD-9-CM: 401–405), diabetes mellitus (ICD-9-CM: 250), hepatitis B (ICD-9-CM: 070.20–070.33), hepatitis C (ICD-9-CM: 070.41, 070.44, 070.51, 070.54, 070.70, and 070.71), HIV (ICD-9-CM: 042), systemic lupus erythematosus (SLE, ICD-9-CM: 710.0), and rheumatoid arthritis (ICD-9-CM: 714) before the index date were considered as comorbidity variables. The main outcome was VZV infection, including chickenpox (ICD-9-CM: 052) and herpes zoster (ICD-9-CM: 053).

Patients who had been diagnosed with chickenpox or herpes zoster between entry into the database and the index date were excluded from the study. Propensity score matching was employed to match the RT cohort to the non-RT cohort with a matching ratio of 1:1. The matching criteria were age, index year, diagnosis year of breast cancer, all comorbidities, and all treatments.

### Statistical analysis

The age group, comorbidity variables, and treatment variables were defined as categorical variables and tested using Chi-square tests to evaluate the distribution of variables between the RT and non-RT cohorts. The difference in average age between the two cohorts was tested using the two-sample Student’s t-test. We calculated the incidence rate (IR) and incidence rate difference (IRD) to assess the absolute risk of VZV infection. Given the mortality of breast cancer, the competing risk regression model was applied to calculate the subhazard ratios (SHRs) of outcome with 95% confidence intervals (95% CIs). Adjusting for age group, comorbidities, and treatments, the multivariable competing risk regression model was used. Stratified analyses of follow-up period were conducted to assess the risk at different time intervals. Statistical analysis was performed using SAS version 9.4 (SAS Institute, Inc., Cary, NC, USA). Statistical significance was accepted at p value < 0.05.

## Results

**Fig 1:**
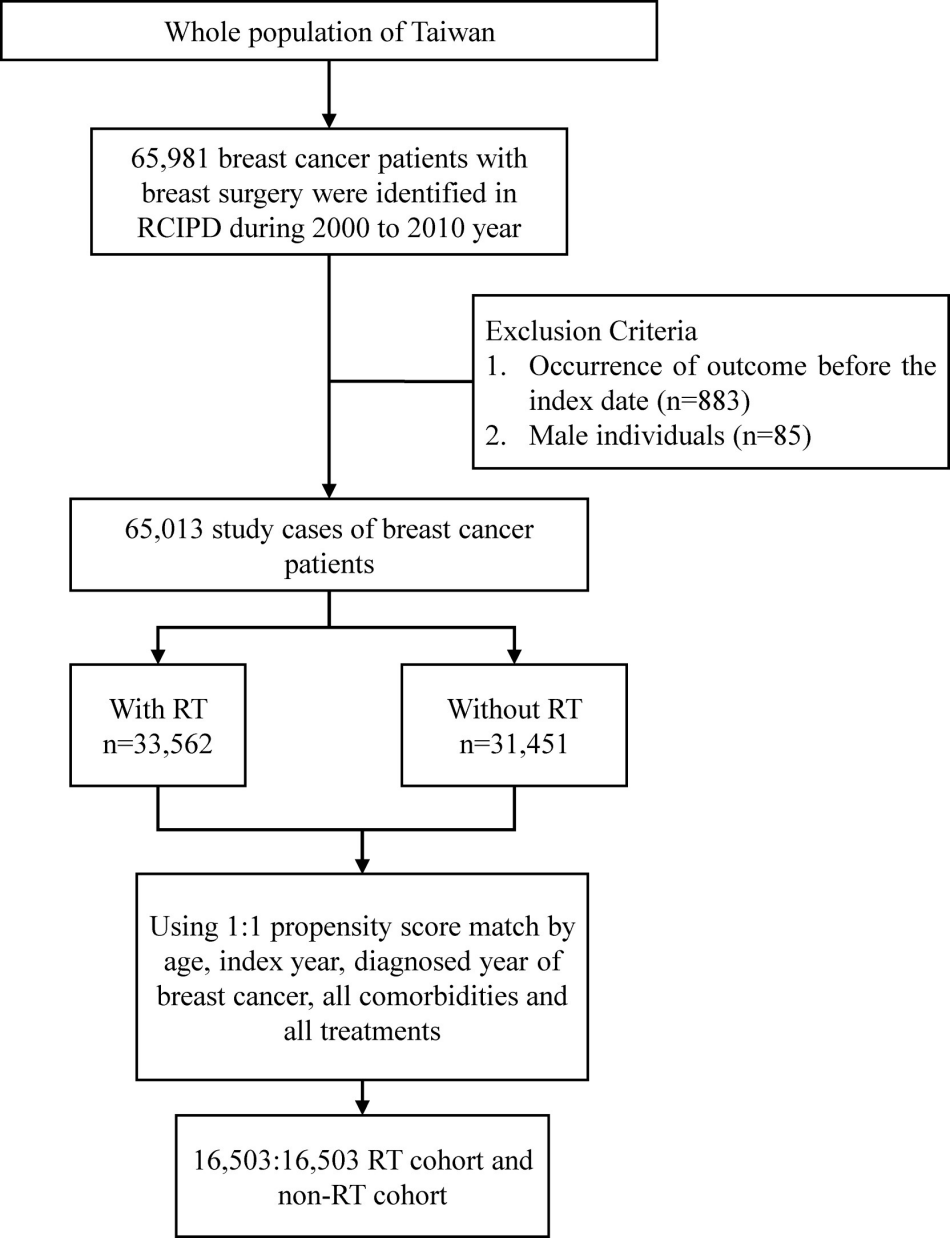
Flowchart for case and control selection. Abbreviations: RCIPD, Catastrophic Illness Patient Database; RT, radiotherapy.

In total, 65,981 patients with breast cancer who underwent breast surgery during 2000–2010 were identified in the RCIPD (Fig 1). After exclusions, 33,562 and 31,451 patients with breast cancer with and without RT treatment, respectively, were identified. The final step of subject selection was the 1:1 propensity score matching. Finally, both RT and non-RT cohorts contained 16,503 patients with breast cancer.

Most patients who underwent RT were in the age range 41 to 65 years (73.7%) (Table 1), as were most of those in the non-RT cohort (74.1%). The mean of age was 53.5 and 54.1 years among the non-RT and RT cohorts, respectively. Age distribution was significantly associated with RT treatment (p = 0.002). The proportions of hypertension, diabetes mellitus, hepatitis C, and SLE comorbidities were higher in the RT cohort than in the non-RT cohort. A significant difference was observed in terms of hypertension between the two cohorts (p = 0.001). In total, 16,503 patients with RT were identified, with 8658 (52.5%) and 11,845 (71.8%) receiving chemotherapy and hormone therapy, respectively. The distribution of chemotherapy treatment between the non-RT and RT cohorts was significantly different (p < 0.001).

**Table 1:** Demographic characteristics and covariates in breast cancer patients with and without radiation therapy.

Variables	Radiation therapy				p value*
	No (N = 16503)		Yes (N = 16503)		
	n	%	n	%	
**Age, years**					0.002
<40	1561	9.46	1433	8.68	
40–49	5365	32.5	4927	29.9	
50–64	6865	41.6	7236	43.9	
>65	2712	16.4	2907	17.6	
Mean(SD)	53.5	11.3	54.1	11.2	<0.001†
**Baseline comorbidity**					
Hypertension	5416	32.8	5696	34.5	0.001
Diabetes mellitus	3015	18.3	3126	18.9	0.12
Hepatitis B	839	5.08	814	4.93	0.53
Hepatitis C	325	1.97	335	2.03	0.69
HIV	1	0.01	1	0.01	0.99
SLE	20	0.12	25	0.15	0.46
RA	62	0.38	59	0.36	0.78
**Treatment**					
Chemotherapy	9249	56.0	8658	52.5	<0.001
Hormone therapy	11950	72.4	11845	71.8	0.20

Abbreviation: SD, standard deviation; HIV, human immunodeficiency virus; SLE, systemic lupus erythematosus; RA, rheumatoid arthritis

*Chi-square test

†Two sample t-test

**Fig 2:**
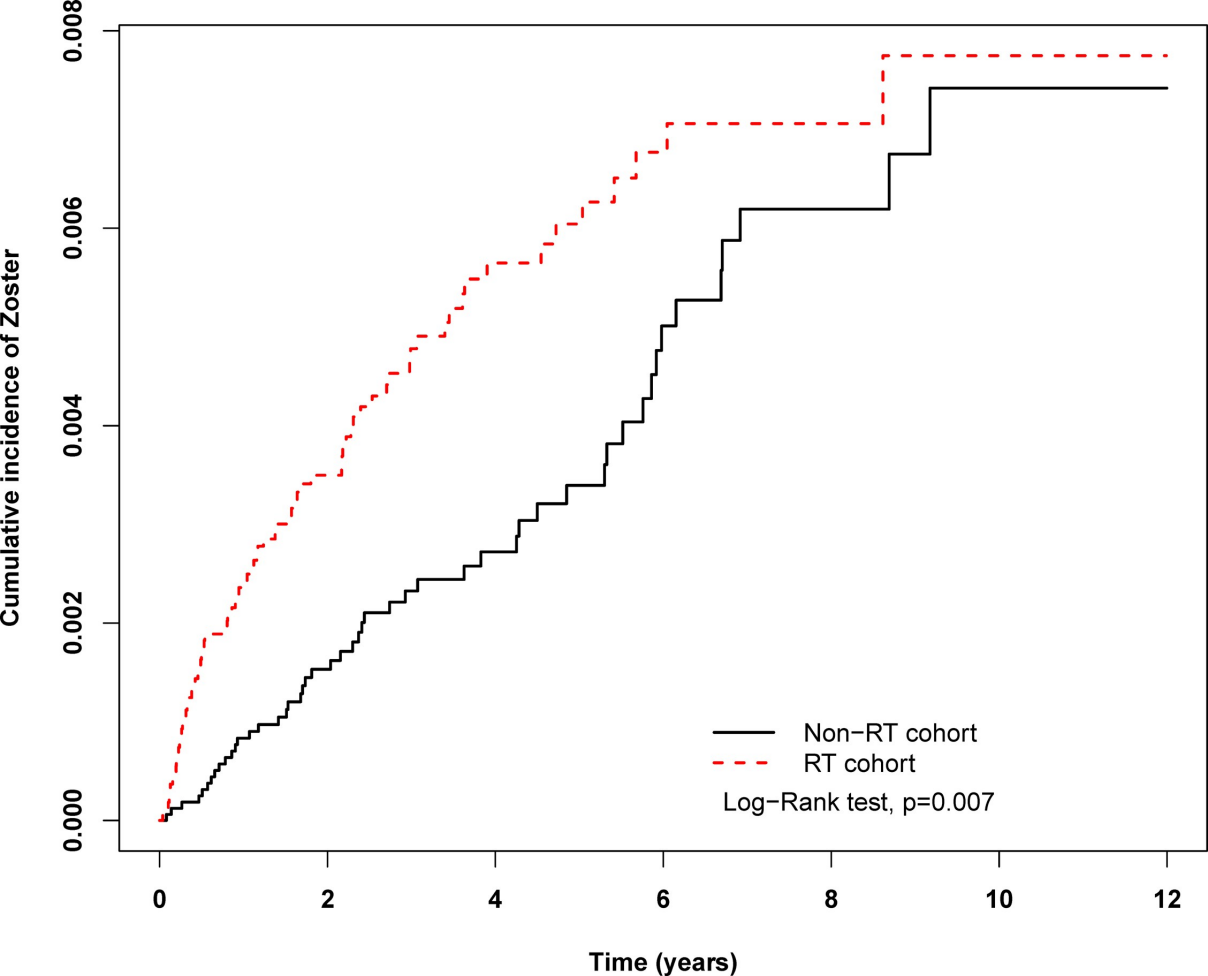
The estimated cumulative incidence of zoster among breast cancer patients with and without radiotherapy by Kaplan-Meier analysis. Abbreviations: RT, radiotherapy.

Using the Kaplan–Meier method, the cumulative incidence of VZV infection in patients with breast cancer was discovered to be significantly higher in the RT cohort than in the non-RT cohort (p = 0.007) (Fig 2). In total, 50 and 77 VZV infection events were noted in non-RT and RT cohorts, respectively (Table 2). Among all variables, only RT was a significant risk factor for VZV infection in the competing risk regression model (adjusted hazard ratio [HR] = 1.51, 95% CI = 1.06–2.16, p = 0.02, IRD = 4.98/10000 person-years). Chemotherapy also had a trend of increasing the risk of zoster virus infection (adjusted SHR = 1.25, 95% CI = 0.88–1.78, p = 0.21).

**Table 2:** Competing risk regression model measured subhazard ratio and 95% confidence intervals of zoster associated breast cancer patients with and without radiation therapy and covariates.

Characteristics	Event no. (n = 127)	Crude				Adjusted		
		SHR	(95% CI)	P value		SHR	(95% CI)	P value
**Radiation therapy**								
No	50	1	reference			1	reference	
Yes	77	1.52	(1.06–2.17)		0.02	1.51	(1.06–2.16)	0.02
**Age, years**								
<40	12	1	reference			1	reference	
40–49	35	0.86	(0.45–1.65)		0.65	0.86	(0.44–1.67)	0.66
50–64	64	1.21	(0.66–2.24)		0.55	1.23	(0.64–2.34)	0.54
>65	16	0.75	(0.36–1.59)		0.46	0.81	(0.34–1.94)	0.64
**Baseline comorbidity**^**#**^								
No	76	1	reference			1	reference	
Yes	51	0.91	(0.64–1.3)		0.62	0.97	(0.64–1.47)	0.89
**Treatment**								
Chemotherapy								
No	58	1	reference			1	reference	
Yes	69	1.28	(0.91–1.82)		0.16	1.27	(0.89–1.8)	0.19
Hormone therapy								
No	35	1	reference			1	reference	
Yes	92	0.98	(0.66–1.44)		0.90	1.01	(0.68–1.5)	0.96

Abbreviation: SHR, subhazard ratio; CI, confidence interval.

Adjusted SHR: adjusted for age, baseline comorbidity and all treatments in competing risk regression model.

^#^with any history of hypertension, diabetes mellitus, hepatitis B, hepatitis C, HIV, SLE or RA.

Zoster incidences were 7.47 and 12.45 per 10000 person-years in the non-RT and RT cohorts, respectively (Table 3). RT patients over 65 years old showed 3.85-fold higher VZV infection risk than non-RT patients from the same age group (95% CI = 1.1–13.4, p < 0.05, IRD = 14.33/10,000 person-years). Furthermore, the highest IRD was 11.09/10,000 person-years in the subgroup of population age over 65 years. In patients without baseline comorbidity, RT treatment increased the risk of VZV infection (adjusted SHR = 1.62, 95% CI = 1.02–2.57, p < 0.05, IRD = 5.75/10,000 person-years). Compared with the patients who received chemotherapy but not RT, the patients who received both RT and chemotherapy had a higher risk of VZV infection (adjusted SHR = 1.68, 95% CI = 1.03–2.73, p < 0.05, IRD = 7.98/10,000 person-years). Among those who did not receive hormone therapy, the RT cohort had a 2.7-fold higher VZV infection risk than the non-RT cohort (95% CI = 1.26–5.71, p < 0.05, IRD = 10/10,000 person-years).

**Table 3:** Incidence rates, subhazard ratio and confidence intervals of zoster among breast cancer with and without radiation therapy in the stratification of age group, baseline comorbidity and treatments.

Variables	Radiation therapy (RT)						IRD	RT VS. Non-RT	
	No (n = 16503)			Yes (n = 16503)				Crude SHR (95% CI)	Adjusted SHR (95% CI)
	Event	Person years	IR	Event	Person years	IR			
**Overall**	50	66924	7.47	77	61868	12.45	4.98	1.52(1.06–2.17)*	1.51(1.06–2.16)*
**Age, years**									
<40	3	6891	4.35	9	6332	14.21	9.86	3.09(0.83–11.4)	3.01(0.81–11.2)
40–49	15	23733	6.32	20	20565	9.73	3.41	1.42(0.73–2.77)	1.41(0.72–2.76)
50–64	29	27042	10.72	35	25901	13.51	2.79	1.14(0.69–1.86)	1.14(0.7–1.86)
≥65	3	9259	3.24	13	9070	14.33	11.09	4.03(1.15–14.1)*	3.85(1.1–13.4)*
**Baseline comorbidity**^**#**^									
No	29	40610	7.14	47	36471	12.89	5.75	1.64(1.03–2.61)*	1.62(1.02–2.57)*
Yes	21	26314	7.98	30	25397	11.81	3.83	1.36(0.78–2.37)	1.35(0.77–2.36)
**Treatment**									
Chemotherapy									
No	24	39000	6.15	34	37000	9.19	3.04	1.39(0.83–2.33)	1.39(0.83–2.33)
Yes	26	27924	9.31	43	24868	17.29	7.98	1.69(1.04–2.75)*	1.68(1.03–2.73)*
Hormone therapy									
No	9	16109	5.59	26	16677	15.59	10	2.73(1.28–5.83)**	2.68(1.26–5.71)*
Yes	41	50815	8.07	51	45191	11.29	3.22	1.25(0.83–1.89)	1.25(0.83–1.89)

Abbreviation: IR, incidence rates, per 10,000 person-years; RT, radiotherapy; IRD, incidence rate difference, per 10,000 person-years; SHR, subhazard ratio; CI, confidence interval.

Adjusted SHR: adjusted for age, baseline comorbidity and all treatments in competing risk regression.

^#^with any history of hypertension, diabetes mellitus, hepatitis B, hepatitis C, HIV, SLE or RA.

* <0.05

** <0.01

The analysis of follow-up period stratification indicated that VZV infection risk was significantly high within 5 month post receiving RT (Table 4). The adjusted SHR of VZV infection were 6.6 (95% CI = 1.51–28.8, p < 0.05, IRD = 32.01/10,000 person-years) and 7.08 (95% CI = 1.64–30.5, p < 0.01, IRD = 35.72/10,000 person-years) in follow-up period less than 3 months and 3–5 months respectively. The median time after radiation to infection was 1.04 years. VZV infection risk causing by RT was not significant after 6 months post-RT.

**Table 4:** Zoster incidence in breast cancer patients with and without radiation therapy at different follow-up time in competing risk regression model.

Follow-up time, months	Breast cancer						IRD	Crude SHR (95% CI)	Adjusted SHR (95%CI)
	Non-RT			RT					
	Event	Person years	IR	Event	Person years	IR			
<3	2	4059	4.93	13	4061	32.01	27.08	6.48(1.46–28.7)*	6.6(1.51–28.8)*
3–5	2	3950	5.06	14	3919	35.72	30.66	7.02(1.6–30.9)**	7.08(1.64–30.5)**
6–11	9	7681	11.72	10	7513	13.31	1.59	1.12(0.46–2.77)	1.11(0.45–2.74)
≥12	37	51234	7.22	40	46375	8.63	1.41	1.1(0.7–1.72)	1.11(0.71–1.73)

Abbreviation: IR, incidence rates, per 10,000 person-years; RT, radiotherapy; IRD, incidence rate difference, per 10,000 person-years; SHR, subhazard ratio. CI, confidence interval.

Adjusted SHR: adjusted for age, baseline comorbidity and all treatments in competing risk regression.

* <0.05

** <0.01.

## Discussion

In this population-based study, we determined that the risk of VZV infection among patients with breast cancer was significantly higher in those who received RT than in those who did not (adjusted HR = 1.51, 95% CI = 1.06–2.16, p = 0.02, IRD = 4.98/10,000 person-years). In the subgroup of patients who received chemotherapy, the patients with RT treatment showed a higher risk of VZV infection than those without RT (adjusted SHR = 1.68, 95% CI = 1.03–2.73, p < 0.05, IRD = 7.98/10,000 person-years). Among the patients over 65 years old, the highest risk was posed by RT (adjusted SHR = 3.85, 95% CI = 1.1–13.4, p < 0.05, IRD = 11.09/10,000 person-years). The VZV infection risk causing by RT was only significant within the first 5 months (adjusted SHR = 6.66, 95% CI = 1.51–28.8, p < 0.05, IRD = 32.01/10,000 person-years in follow-up period less than 3 months and adjusted SHR = 7.08, 95% CI = 1.64–30.5, p < 0.01, IRD = 35.72/10,000 person-years within 3–5 months). Based on our findings, we conclude that RT contributes to the risk of VZV infection in patients with breast cancer. The RT-induced VZV infection is likely to be seen within the first 5 months, and later occurrences are less clearly related to the therapy.

The activation of VZV is associated with compromised immunity due to conditions such as HIV infection [11], diabetes mellitus [12], transplantation [13], and malignancies [14]. For cancer-related treatment, chemotherapy is believed to be a risk factor for VZV infection [15]. Masci et al. [16] analyzed 623 patients in a single institution and observed that the risk of developing VZV infection was 13 times higher among patients with breast cancer who underwent chemotherapy than among the general population.

The possible mechanism of RT induced VZV infection has been well discussed in the review article written by Ramirez-Fort et al [17]. They pointed out that radiation may eradicate the cellular immune component in the irradiated region, which could contribute to the susceptibility of primary viral contraction and/or reactivate existing latent virus (from previous infections). The paper also noted that the serological testing for human herpes virus should be considered before RT to alert the clinician of the advisability of antiviral therapy during and after the RT course to prevent reactivated infection and also to differentiate between infection and radiation necrosis. However, few studies have confirmed RT as a risk factor for VZV infection, especially by direct comparison between the RT and non-RT group. Most studies have focused on Hodgkin disease, probably because aggressive treatments such as extended-field RT may induce a higher incidence of VZV infection [18–20]. Reboul et al. [19] observed that the risk of VZV infection is approximately 2-fold higher in patients who undergo extended-field RT (23.8%) than in those who undergo limited-field RT (11.1%). One study has evaluated patients with breast cancer treated with radiation. Dunst et al. [9] retrospectively analyzed 1155 patients with breast cancer who received postoperative irradiation either after mastectomy (n = 961) or after breast preservation (n = 194) in a single institution. The dose of RT varied from 44 to 58 Gy in 1.8 to 2 Gy fractions. Higher frequency of zoster infection was observed (3.7%) in this cohort compared with the general population (0.25%–0.35%). However, the role of radiation in zoster infection could not be determined because all patients had received RT. The strengths of our population-based study are that it involved a direct comparison (RT versus non-RT), a large sample (65,981), and adequate controls for determining comorbidities.

However, this study was subject to limitations. First, the NHIRD does not provide detailed information on potential confounding factors such as body mass index, smoking habits, alcoholic consumption, family history, or cancer stage. Second, we were unable to eliminate the impact of unobserved confounders because a retrospective study was conducted. Third, clinical data prior to 1995 was not available because the NHI of Taiwan was not implemented before this time. Therefore, we were unable to separately analyze the subgroup of patients who had ever (lifetime) received the related vaccination or experienced previous episodes of VZV infection. Moreover, the NHIRD provides no information on RT dose level, energy level, technique, target volume, or distribution. This precluded us from performing any analysis involving these variables.

## Conclusion

In conclusion, our study was the first to provide evidence using a large-scale nationwide cohort study to determine the role of RT in VZV infection among patients with breast cancer. Our finding demonstrated that the risk of VZV infection is higher in RT cohort than in non-RT cohort. Based on our study, we suggest additional attention to older breast cancer patients (>65 years old) and/or those who receive chemotherapy. Regular clinical follow-up and additional serological testing in the first 5 months after RT are recommended to ensure early diagnosis and appropriate treatment and thus prevent serious sequelae.
